# Numerical Low-Back Booster Analysis on a 6-Year-Old Infant during a Frontal Crash Test

**DOI:** 10.1155/2018/2359262

**Published:** 2018-07-16

**Authors:** I. L. Cruz-Jaramillo, C. R. Torres-San-Miguel, O. Cortes-Vásquez, L. Martínez-Sáez

**Affiliations:** ^1^Instituto Politécnico Nacional, Sección de Estudios de Posgrado e Investigación, Unidad Profesional “Adolfo López Mateos”, Escuela Superior de Ingeniería Mecánica y Eléctrica Zacatenco, Edificio 5, 2° Piso Col. Lindavista, 07738 México City, Mexico; ^2^Universidad Politécnica de Madrid, Instituto Universitario de Investigación del Automóvil, Campus Sur UPM, Carretera de Valencia A-3, km. 7, 28031 Madrid, Spain

## Abstract

This work studies descriptively the Head Injury Criterion (HIC) and Chest Severity Index (CSI), with a finite element model of the Hybrid III dummy type, for six-year-old subjects in a frontal vehicular collision, using the low-back booster (LBB) passive safety system. The vehicle seats and the passive safety systems were modelled in CAD (computer aided design) software. Then, the elements were analysed by the finite element method (FEM) in LS-DYNA® software. The boundary conditions were established for each study, according to the regulations established by the Federal Motor Vehicle Safety Standard (FMVSS), following the FMVSS 213 standard. The numerical simulations were performed during an interval of 120 ms and recording results every 1 ms. In order to analyse the efficiency of the system, the restraint performance of the LBB system is compared with the restraint configuration of the vehicle safety belt (VSB) only. The obtained injury criteria with the LBB system shows its ability to protect children in a frontal collision. The analyses allow obtaining the deceleration values to which the dummy head and chest was subjected. Of the studies herein performed, Study I: VSB obtained a HIC_36_ of 730.4 and CSI of 315.5, while Study II: LBB obtained a HIC_36_ of 554.3 and CSI of 281.9. The outcome shows that the restraint efficiency of each studied case differs. Used materials, the attachment system of the LBB, and the belt restraint system properly placed over the infant trunk are the main factors reducing the injury criteria rate.

## 1. Introduction

Annually, more than 260,000 children die worldwide as result of traffic collisions; it is also estimated that up to 10 million of them suffer nonfatal injuries. Trauma caused by traffic accidents are the second cause of death for children aged 5–14 years. The 22.3% of children who died during 2004 from 0 to 14 years old were involved in traffic accidents, of which the ones aged 5–9 years showed the highest mortality rate [1]. In Mexico, between 2000 and 2010, 17,700 children under the age of 15 have died in traffic accidents [2]. In 2011, traffic accidents in Mexico City became the third cause of death for children aged 5 to 9 years, as well as the fourth for children aged 10 to 14 years [3].

In 1972, the first Federal Safety Standard for children occupying vehicles FMVSS 213 (Federal Motor Vehicle Safety Standard) was issued, which specifies the requirements for infant seats to be marketed in the United States of America [4].

Severe injuries on the pelvis, shoulder, thorax, neck, and head have often occurred in frontal collisions. The neck usually experiences the inertial load generated by the head; during the initial phase of a crash, more restraint is applied in the lower neck, while the head is normally subjected to a horizontal translational displacement relative to the torso, inducing neck extension movements in frontal collisions [5]. Such movement generates high traction loads on the neck during the main horizontal deceleration of the head. The previous phenomenon occurs when no head contact exists with external objects like the backs of front seats.

The neck is exposed to significant mechanical loads when the natural range of neck extension and flexion is overpassed, causing elongation and tearing in different ligaments, as well as even the dislocation of the neck's articulations [6].

On the other side, chest injuries can range from rib fractures to even more severe injuries that cause internal organs to collide with internal body walls, producing abdominal bruises, wounds, and scratches. Lesions of the thoracic aorta, of the small intestine, or even mesentery, occur when the body abruptly stops while the interior organs and tissues continue to move forward by inertia, causing aortic twisting or tearing of intestinal loops at their mesenteric insertion [7].

The probability to suffer head injuries is calculated using HIC (Head Injury Criterion). The latter is obtained by calculating the resulting linear acceleration of the head's centre of gravity, and it is measured in units of the earth's gravity acceleration (*g*) [8]. HIC does not take into account factors such as the rotational acceleration of the skull or any effect on the location of the impact on the head. A HIC value of 1000 is considered as the threshold for brain lesions [9].

The FMVSS 208 establishes that in order to discard chest damage, the maximum acceleration on the chest must be below 60*g* in a time interval of 3 ms (CLIP3M) and a CSI (Chest Severity Index), calculated as the HIC but over the chest, value lower than 1000 [10].

The American Association of Paediatrics recommends the usage of a low-back booster (LBB) for children aged between 4 and 8 years or weighing 40–80 lb (18–36 kg) [11]. The Hybrid III 6-year-old dummy selected for this study fits well within these ranges of age and weight.

This research is intended to quantify the performance of a LBB system and to validate the numerical models with experimental tests performed by Hagedorn and Stammen [12].

## 2. Methods

In order to quantify the differences between HIC and CSI when implementing the LBB and vehicle seat belt (VSB), two analysis scenarios were proposed and simulated with the LS-Dyna v. 9.71 software. Both analyses were performed with the Hybrid III 6-year-old FEM dummy model. The seat belt was modelled to perform the restraint analysis in both situations: VSB restraint only and VSB with the LBB under the dummy. The LBB Evenflo®, belonging to groups 2 and 3 (adjustable 3–11 years or 18–49.8 kg), was used to design the LBB system in CAD software.

**Figure 1:**
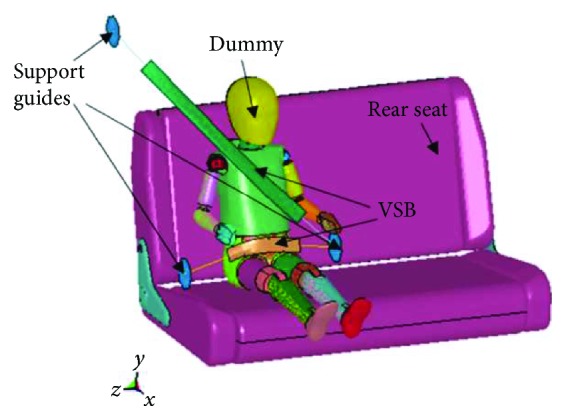
Study I (VSB).

**Figure 2:**
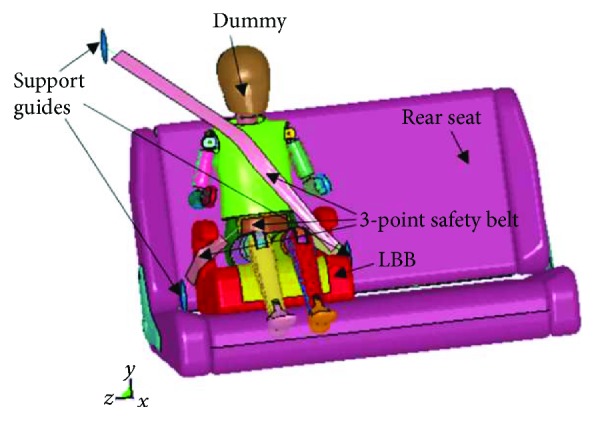
Study II (LBB).

The analyses were carried out with a crash speed of 48 km/h (13.34 m/s) as indicated by the FMVSS. The scenarios were done by implementing 2 passive safety systems (VSB and LBB):

- Study I (VSB): the dummy was placed on the back seat of a sedan vehicle and was restrained with just the seat belt implemented nowadays (Figure 1).
- Study II (LBB): the dummy was seated on the LBB and was secured with the 3-point safety belt included with the vehicle (Figure 2).

### 2.1. Designs and Materials

The vehicle rear seat was designed in CAD software accomplishing the approximate size of the rear seats of a model 2008 Honda Fit® vehicle.

The LBB is mounted on the rear seat as indicated in its user manual and the vehicle's user manual. The rear seat of the vehicle is composed of two materials: the steel bracket and the foam. A plastic holder is used as a joint between the seat backrest part and the seat cushion part.

In the same way, the LBB was designed with the measurements of the LBB Evenflo. It consists of structural material as well as foam. The rear seat with the LBB was assembled by CAD. Each study case is meshed by an Arbitrary Lagrangian-Eulerian (ALE) mesh, because it is a self-adjustable meshing method [13]. A mesh of 8 mm in size composed of 3D tetrahedral elements is computed and generated using the HyperMesh® V.14.0 software.

In order to accurately model the LBB structural material, polypropylene was selected. Polypropylene has the following mechanical properties: density of 9 × 10^−7^ kg/mm^3^, Young's modulus of 1.35 GPa, elastic limit of 0.036 GPa, and Poisson's ratio of 0.3 [14].

For the padding of the seat and LBB, the DAX 55 foam was chosen. This foam has the following mechanical properties: density of 3.5 × 10^−8^ kg/mm^3^, Young's modulus of 5 × 10^−5^ GPa, and Poisson's ratio of 0.31 [15].

For the polypropylene and FAX 55 foam, the data presented above is to be loaded in LS-DYNA [16].

The mechanical properties of the steel bracket of the rear seat are: density of 7800 kg/mm^3^, Young's modulus of 210 GPa, elastic limit of 0.6 GPa, Poisson's ratio of 0.3, and tangent modulus of 0.3 GPa.

**Figure 3:**
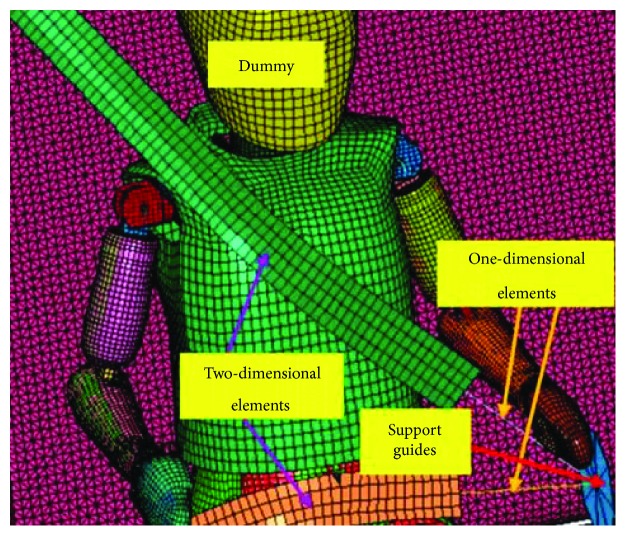
Design of the safety belts.

The design of the 3-point safety belt was made according to the specifications of the selected model of the belt, which is 4.7 cm wide by 1 mm thick. The design of the belts was performed with the BELTFIT tool in the LS-DYNA software. The belt placement was performed according to the National Highway Traffic Safety Administration (NHTSA) standard (Figure 3). The design of the belts considered one-dimensional and two-dimensional elements (Figure 3).

Section and material for the one-dimensional elements were assigned with the predetermined seatbelt configuration in LS-DYNA. This material has a linear density of *λ* = 5.97 × 10^−4^ kg/mm [17].

The two-dimensional elements were assigned a shell-type section with a thickness of 1 mm. Also, these elements were endowed with an elastoplastic-type material, with a piecewise linear plasticity behaviour and the mechanical properties of nylon: density of 1 × 10^−6^ kg/mm^3^, Young's modulus of 5.333 GPa, elastic limit of 0.08 GPa, and Poisson's ratio 0.3.

Curves of load and unload, representing the axial force as a function of the strain of the safety belt, were obtained by Dhole et al. [18]. The seat belts' anchorage guides and fixation points were approximately placed according to height and distances for the considered sedan vehicle.

In the case of the LBB system, the lap belt portion of the VSB has been upgraded to include 3 lengths of two-dimensional elements allowing the restraint of the LBB by the VSB.

### 2.2. Boundary Conditions

The initial speed of the system is 13.34 m/s along the “*x*” axis. In the “*y*” direction corresponding with the vehicle vertical direction, the only force acting is gravity (“−*y*” axis), with a constant value of 0.00981 mm/ms^2^. The deceleration curves in the “*x*” direction are taken from FMVSS regulation and are introduced in the software.

Contact boundary conditions were selected as automatic in the software for explicit simulations. In the tangential direction to the plane of the contact, the resulting stresses are due to friction between parts, and the stresses are defined by static and dynamic friction coefficients of 0.3 and 0.2, respectively [19]. The nodal elements (foam-LBB) were linked for them to behave like one body.

## 3. Results

**Figure 4:**
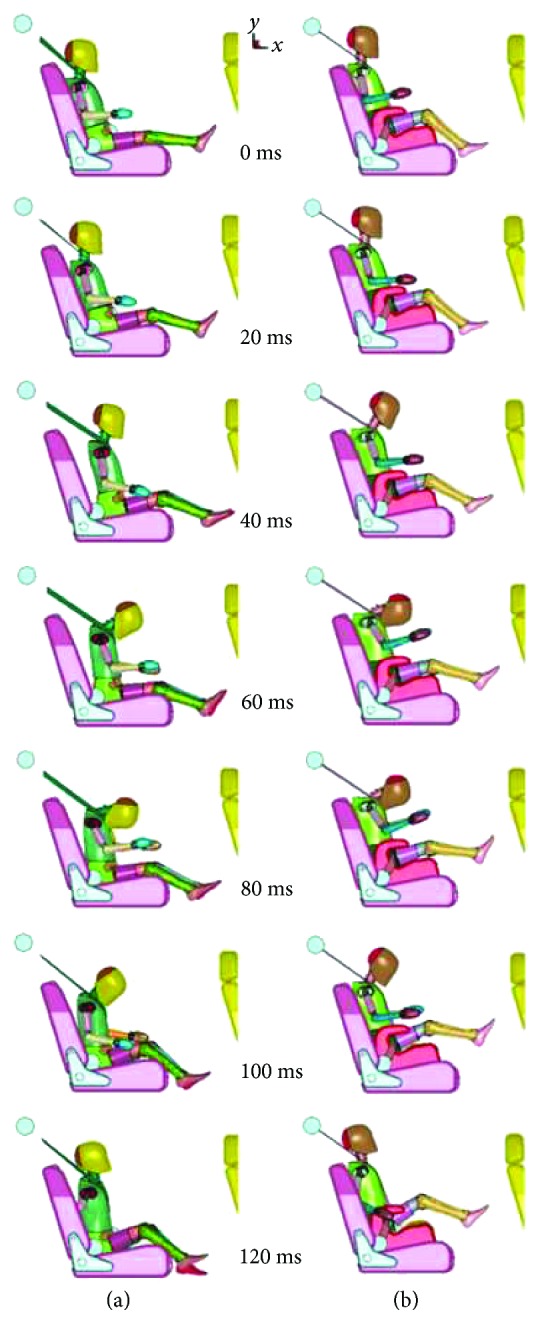
Dummy kinematics results: (a) VSB and (b) LBB.

Numerical simulations were performed in a range of 120 ms, recording results every 20 ms (Figure 4). In the next paragraphs, we present the results for each anatomic part analysed from the dummy (head and chest).

### 3.1. Head

**Figure 5:**
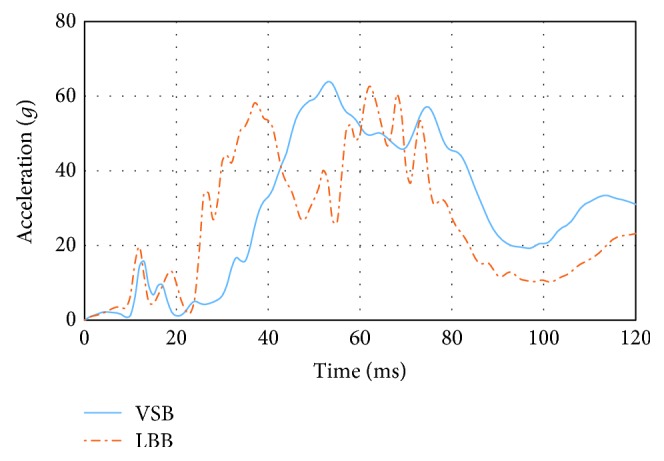
Head resultant acceleration.

Figure 5 shows the dummy head resultant acceleration for both LBB and VSB systems, which were obtained by an accelerometer sensor type located in the centre of gravity of the dummy head used in the simulations.

Figure 5 shows that the head resultant acceleration ramps up earlier for the LBB than for the VSB, due to a better connection of the dummy trunk for the LBB than in the case of the VSB. The same tendency can be observed for the resultant acceleration of the dummy thoracic spine analysed from the latter. This fact allows for a better restraint for the dummy during the earliest stages of the crash producing a wider restraint phase with lower maximum values at the end when the LBB system is used.

The lower value of HIC_36_ is presented for the LBB system, with a value of just 554.3 and a maximum peak head resultant acceleration of 62*g*, while for the VSB system, the same injury criteria increment is 730.4 and 65*g*.

### 3.2. Thorax

**Figure 6:**
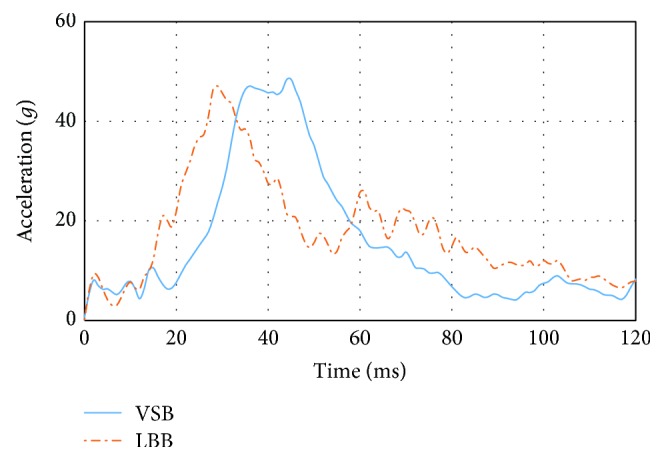
Thoracic spine resultant acceleration (*g*).

The analyses done in Section 3.1 were also reproduced for the chest, by placing the numerical accelerometer in the thoracic spine of the dummy's thorax. The position of the thoracic spine accelerometer in the dummy model replicates the position in the current physical dummy. Although the thoracic spine acceleration is not an appropriate parameter to derive injury criteria for the chest, it has been used in this article to obtain references to the thoracic restraint capabilities of systems studied. The resultant thoracic spine deceleration value was obtained in the crash simulation, and later it was used to calculate the CSI. For CSI calculation, the same formula as the one used for the HIC has been employed replacing the head centre of gravity resultant acceleration by the thoracic spine resultant acceleration. Even the 3 ms cumulative resultant thoracic spine acceleration criterion was also obtained as FMVSS 213 was established. Thoracic spine resultant acceleration for both LBB and VSB can be observed in Figure 6.

LBB generated a CSI of 281.9 and a thoracic spine acceleration 3 ms clip of 44.36, while VSB yields to CSI values of 315.5 and thoracic spine acceleration 3 ms clip of 45.23, respectively. The area under the curves displayed in Figure 6, between 20*g* and 40*g*, shows an indication of the energy absorbed by the dummy trunk. It can be observed in the lower area for the LBB system compared to the VSB system. This fact produces a better restraint for the dummy pelvis area of the LBB system and includes the thoracic spine because the pelvis is linked to the lower portion of the thoracic spine through the lumbar spine.

**Figure 7:**
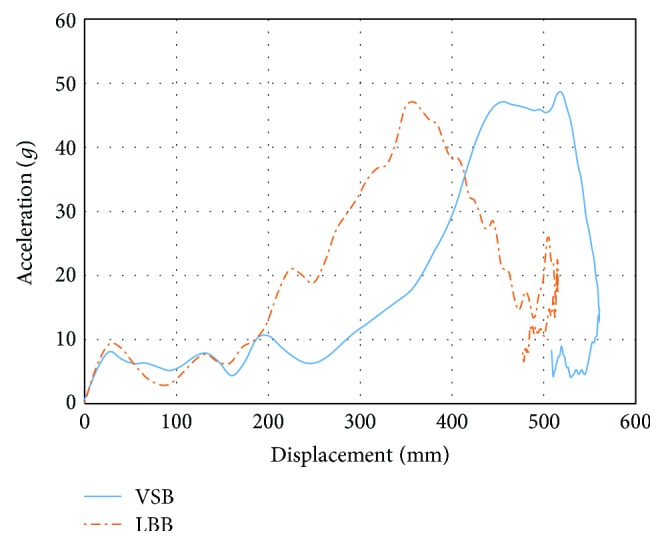
Effectiveness of the LBB and VSB restraint.

Figure 7 shows a comparison of the longitudinal acceleration of the chest in the two tests. The maximum thoracic spine displacement of LBB is lower than VSB and the highest energy absorption was generated in the last 300 mm.

## 4. Discussion

To validate the numerical analyses herein performed, a comparison of the peak values of the obtained uniaxial decelerations of the head and thorax, for the HIC_36_ and for the thoracic spine acceleration 3 ms clip, with experimental tests, is shown in Table 1 [12].

In Table 1, the HIC_36_ results approximate the experimental results with an error of less than 7.7% in the worst case. Also, it can be inferred that the principal reason for this error is because of the difference in dimension, geometry, and mass in the experimental and numerical seats that were used in this study. Likewise, in both restraint systems analysed, a higher HIC_36_ is generated when VSB is implemented with respect to the use of the LBB system.

## 5. Conclusion

The LBB system generates the smallest HIC_36_, because the belt properly holds the dummy when its sitting height increases, properly fastening the belt over the children's shoulder and pelvic area. This allows a child to benefit from the protection provided by three-point seat belts in frontal impacts by properly distributing the loads on the pelvis, thorax, and the shoulder during a frontal crash. Likewise, this reduces the risk of child movement by inertial force and the seat belt forces over the trunk are lower, reducing the neck moment, decreasing the HIC.

In this study, the best protection level obtained with the LBB system is based on two fundamental factors: a good anchorage behaviour to the vehicle and the improvement of the fit of the seat belt on both the pelvic and shoulder area of the child.

There is, indeed, a clear need to improve a booster seat design to achieve higher levels of protection for children between 4 and 8 years old during frontal impacts. This could be through the usage of a fully rigid anchorage system and the adjustable height of the LBB system to allow the seat belt of the vehicle to fit properly to the child depending on his age and anthropometry.

Due to the high cost and time involved in developing the experimental tests herein presented, it is feasible to perform them numerically in order to simulate nonlinear physical phenomena, in order to obtain approximate values with respect to the experimental tests, allowing the minimisation of costs. Numerical tests also offer the possibility of modifying variables to carry out new analyses. In addition, numerical tests also allow simulating side and rear impact scenarios with the LBB.

## Figures and Tables

**Table 1:** Comparison of numerical and experimental results.

Numerical results		
Test 1		
HIC_36_ = 730.43	TSA3MSC = 45.23*g*	
HAx = 58	HAy = 51	HAz = 7.4
TSAx = 46	TSAy = 10	TSAz = 6
Test 2		
HIC_36_ = 554.3	TSA3MSC = 44.36*g*	
HAx = 46	HAy = 53	HAz = 15
TSAx = 45	TSAy = 12	TSAz = 6.5

Experimental results by Hagedorn and Stammen [12]		
Test 10		
HIC_36_ = 801	TSA3MSC = 44.3*g*	
HAx = 65	HAy = 51	HAz = 6.5
TSAx = 40	TSAy = 20	TSAz = 14
Test 6		
HIC_36_ = 594	TSA3MSC = 50.8*g*	
HAx = 51	HAy = 47	HAz = 7
TSAx = 50	TSAy = 17	TSAz = 5

HAx, head acceleration axis *x*; HAy, head acceleration axis *y* vertical; HAz, head acceleration axis *z*; TSAx, thoracic spine acceleration axis *x*; TSAy, thoracic spine acceleration axis *y* vertical; and TSAz, thoracic spine acceleration axis *z*.

## Data Availability

The data used to support the findings of this study are available from the corresponding author upon request.
